# Isothermal microcalorimetry measures UCP1-mediated thermogenesis in mature brite adipocytes

**DOI:** 10.1038/s42003-021-02639-4

**Published:** 2021-09-21

**Authors:** Muhammad Hamza Bokhari, Carina Halleskog, Alice Åslund, Nathalie Boulet, Eva Casadesús Rendos, Jasper Martin Anton de Jong, Robert Csikasz, Ez-Zoubir Amri, Irina Shabalina, Tore Bengtsson

**Affiliations:** 1 Department of Molecular Biosciences, The Wenner-Gren Institute, Stockholm University, Stockholm, Sweden; 2 Institut des Maladies Métaboliques et Cardiovasculaires, INSERM/Université Paul Sabatier, Toulouse, France; 3 Department of Comparative Medicine, Yale School of Medicine, New Haven, CT USA; 4 Université cote d’azur, CNRS Inserm IBV, Nice, France

**Keywords:** Biological techniques, Fat metabolism

## Abstract

The activation of thermogenesis in adipose tissue has emerged as an important target for the development of novel anti-obesity therapies. Using multi-well isothermal microcalorimetry, we have demonstrated that mature murine brown and brite adipocytes produce quantifiable heat upon β_3_-AR stimulation, independently of any anaerobic mechanisms. Additionally, in brite adipocytes lacking UCP1 protein, β_3_-AR stimulation still induces heat production, albeit to a much lower extent than in their wildtype counterparts, suggesting that UCP1 is an essential component of adrenergic induced thermogenesis in murine brite adipocytes exvivo. Similarly, we could observe an increase in heat production in human-derived adipocytes (hMADS) upon β-AR stimulation. Collectively, these results establish the use of isothermal microcalorimetry as a sensitive and accurate technique for measuring thermogenic responses in intact mature brite adipocytes from murine and human origin.

## Abstract (web summary)

Bokhari et al. demonstrate mature murine brown and brite adipocytes produce quantifiable heat with β3-AR stimulation. They indicate that the essential component of this mechanism is UCP1 by using adipocytes lacking the protein.

## Introduction

Over the past three decades, excess caloric consumption and a sedentary lifestyle have led to an unprecedented increase in the global prevalence of obesity. The discovery of novel weight-loss treatments is therefore essential to aid in combating this epidemic disease. Thermogenic activation of brown adipose tissue has been shown to lead to improvements in various metabolic parameters associated with overweight^1^. It is therefore not surprising that the cells within these tissues have emerged as valuable drug targets for novel anti-obesity drugs.

Adipose tissue can be divided into several functionally and morphologically distinct subtypes. Of these, brown adipose tissue is a key site for non-shivering thermogenesis. The thermogenic capacity of brown adipose tissue is largely attributed to UCP1, a protein embedded within the mitochondrial membrane, that when activated, results in the dissipation of the electrochemical gradient across the mitochondrial membranes leading to increased substrate oxidation and consequently, the production of heat^2^. There also exist other populations of UCP1-containing adipocytes that are molecularly distinct but like brown adipocytes, exhibit thermogenic properties to varying degrees^3,4^. These are found within white adipose tissue depots and are termed as brite^4^ or beige^5^. It has been suggested that these adipocytes can potentially increase thermogenesis by utilizing UCP1-independent mechanisms^6–8^. Since humans have a low amount of UCP1 containing adipocytes and the proposition that these resemble more closely brite adipocytes in mice^9^, these cells are an attractive drug target for increasing thermogenesis to counteract the onset of obesity.

While activation of thermogenesis in human brite adipocytes is hypothesized to result in beneficial metabolic effects, there remain substantial challenges in developing in vitro methods to screen for potential activators of this phenomenon. Oxygen consumption is notoriously difficult to measure in mature adipocytes; conventional plate based respirometry is performed primarily in cultured cells grown from adipocyte precursors but is subject to many pitfalls as reviewed by Keipert et al.^10^. Other methods allow for the measurement of oxygen consumption in mature cells, but either lack in throughput or can induce artefacts into the measurements as the cells are constantly exposed to shear forces due to stirring for the oxygenation of medium.

Adipocytes grown in culture, in contrast to their freshly isolated mature counterparts, have a lower lipid content, a lower thermogenic capacity coupled with a high rate of ATP production^11^. Furthermore, it has been demonstrated that freshly isolated mature adipocytes display a distinct molecular signature different to that of cells grown in culture^12^. Respirometric measurements in cultured brite adipocytes therefore might not fully represent the physiological responses in mature cells. These discrepancies become evident in brown adipocytes, where adrenergic stimulation induces cellular respiration in cultured^13^ but not mature cells derived from UCP1 KO animals^14^.

Isothermal microcalorimetry is a technique that measures direct heat flow from a biological sample over a period of time. The samples are placed in wells on top of a thermopile, a collection of a series of thermocouples that respond to changes in heat production by the generation of a given potential difference. By comparing the voltage induced to a reference standard generated using a thermodynamically defined chemical reaction, the exact amount of heat being produced in the biological sample can be calculated. Older versions of this technique has been used in the past to measure noradrenaline stimulated heat production in mature brown adipocytes isolated from rats^15^ and hamsters^16^, but has been of limited use due to lower sensitivity, low throughput, and the requirement of large sample material.

Modern isothermal microcalorimeters however are optimized for higher sensitivity, high-throughput analysis of low-volume biological samples. They do not require the cells to be adherent or subject to constant stirring for the oxygenation of the medium. Additionally, this technique is extremely sensitive in detecting minute temperature differentials making it an optimal for use with mature brite adipocytes with a low UCP1 content. We have thus demonstrated the use of this technique in freshly isolated murine brite adipocytes highlighting the distinct importance of UCP1 in adrenoceptor mediated thermogenic activity.

## Results

### Methodological overview of isothermal microcalorimetry

**Fig. 1:**
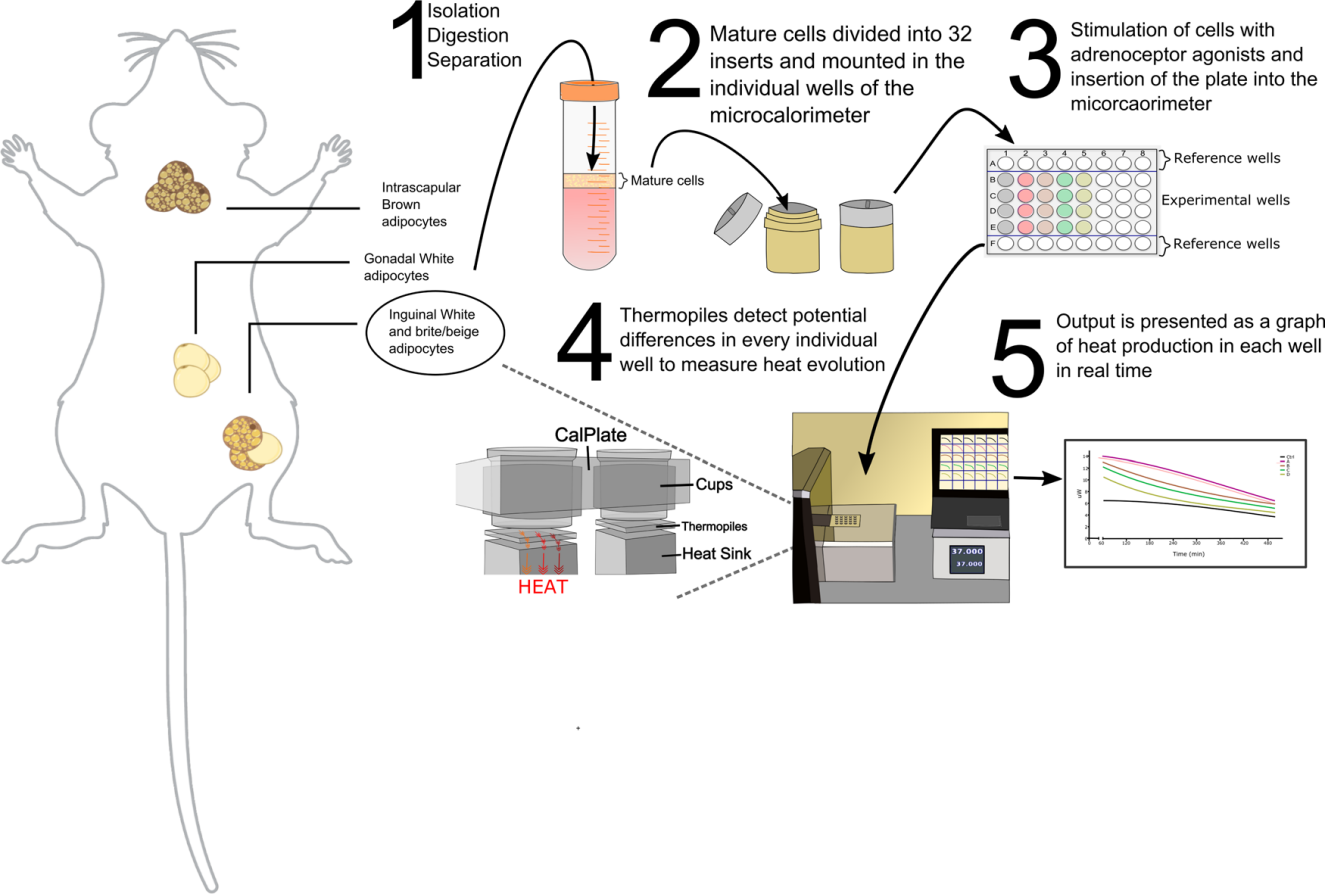
Methodological overview of isothermal microcalorimetry. A brief methodological overview showing the adipocyte isolation protocol and the subsequent measurement of heat production in the isothermal microcalorimeter. Figure created in part with with BioRender.com.

Figure 1 represents a methodological overview of the experimental protocol used for the isolation and the subsequent measurement of agonist induced heat production in mature adipocytes. Briefly, mature adipocytes were isolated from brown and inguinal white fat pads of mice and subsequently plated in plastic inserts containing medium. These inserts were not filled completely so as to allow access to air for medium oxygenation. The inserts were then placed in titanium wells and the lid sealed to stop further atmospheric gaseous exchange from occurring. Thereafter, the wells were placed in the sample holder and set inside the isothermal measurement chamber for the calculation of heat flow between each individual titanium well and semiconductor thermopiles. The thermopile potential is proportional to the heat flow and is used to calculate the heat evolved from the samples. The temperature within the isothermal measurement chamber was set to 37 °C for all experiments. As per the manufacturers reference, the short-term signal noise is below 50 nW. As this system allows for the measurement of direct heat generation in floating cells, it is ideal for measuring thermogenic responses in brown and brite adipocytes ex vivo. A more thorough experimental protocol detailing the use of the machine is provided in the “Methods” section.

### Isothermal microcalorimetry allows for long term measurement of basal heat production in mature inguinal brite adipocytes

**Fig. 2:**
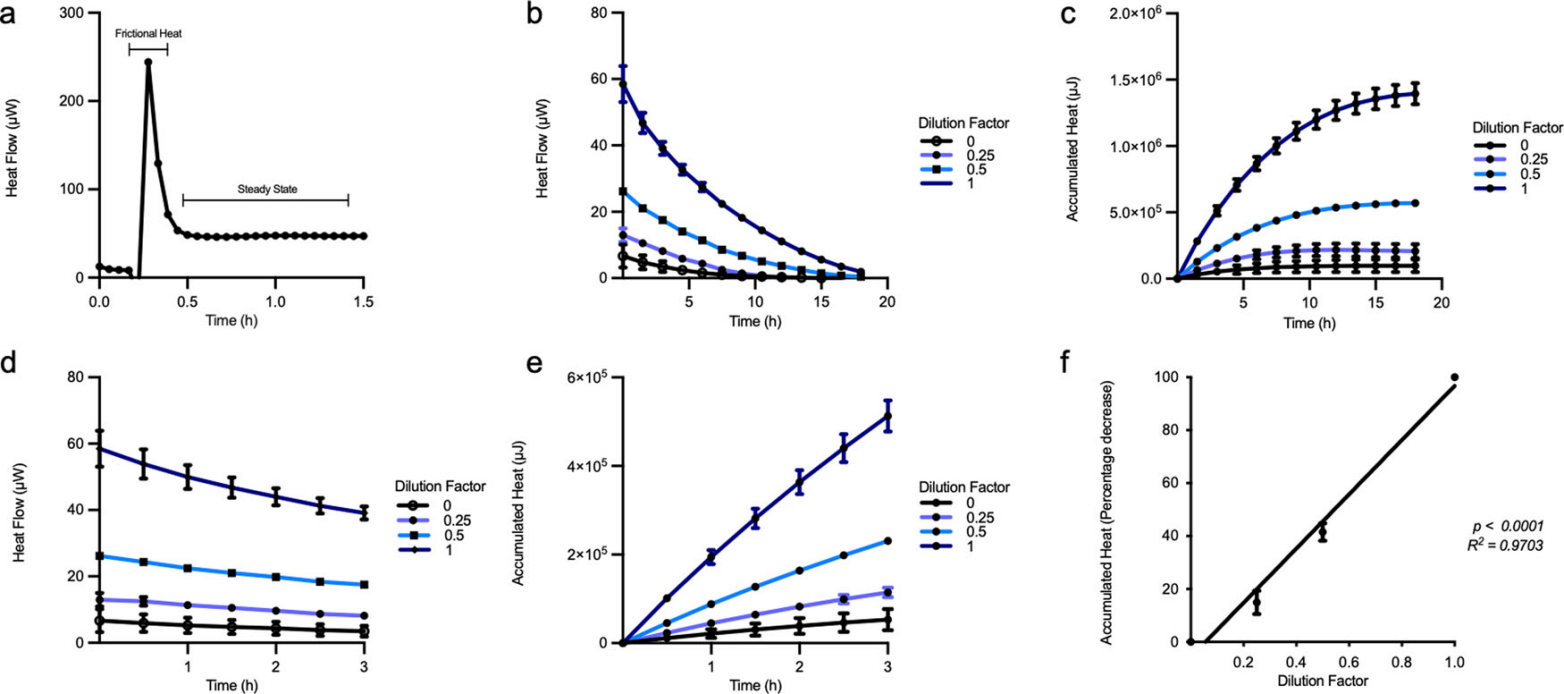
Isothermal microcalorimetry allows for long term measurement of basal heat production in mature inguinal brite adipocytes. **a** Representative trace of the heat output in one microwell in the isothermal microcalorimeter; frictional heat output is shown as indicated; the steady state indicates the region used to calculate cumulative heat production. **b**, **d** Heat flow in μW. **c**, **e** Accumulated heat in μJ recorded from wells in triplicates containing serially diluted isolated mature brite adipocytes for 3 and 18 h from one representative preparation. **f** Normalized average accumulated heat (corrected for wells containing only medium) from wells containing isolated mature brite adipocytes over 1 h expressed as a function of dilution factor from four different preparations plated in triplicates from four independent adipocyte isolations (*n* = 4). The curves show mean value $\pm$ SEM. Linear regression analysis was performed to calculate the *R*^2^ and *P* value.

Figure 2a shows a real time trace of heat production within one singular well containing isolated mature inguinal white adipocytes. The first section of the graph shows a spike in overall heat output as a consequence of frictional heat generated upon setting the titanium wells inside the measurement chamber. Therefore, for all subsequent experiments, we have chosen to present data upon the establishment of a thermal equilibrium so as to discount possible artefacts due to friction; this region of the graph exemplified by the portion of the trace labeled as the “steady state”. In addition, all recorded values were normalized to a baseline value collected at the end of the experiment, where the rate of heat flow was minimal to account for possible well specific differences.

To validate whether the heat production as measured by the machine varies linearly with the concentration of the cellular suspension present in each well and to demonstrate the sensitivity and dynamic range of the instrument, we performed an assay wherein mature adipocytes were isolated from inguinal white adipose tissue depots and then subsequently serially diluted and distributed in the 48-well plate.

Utilizing the maximal capabilities of the available instrumentation, we were able to record heat exchange in our adipocyte preparations for over 20 h (Fig. 2b, c). However, to accurately represent differences in acute heat production, we chose to perform subsequent calculations using the data from the first hour after the cells had stabilized and the thermal equilibrium of the system was re-established (Fig. 2d, e).

We observe a strict linear relationship between the number of cells present within each well and the amount of heat released. This effect was reproducible in a total of four experiments when for each experiment, the measurements were normalized to the accumulated heat production in undiluted cells (Fig. 2f). Our data highlights the capacity of the instrument to discern minute changes in heat production due to differences in cellular amounts with a high sensitivity.

### Brown adipocytes as a classical model to demonstrate thermogenesis

**Fig. 3:**
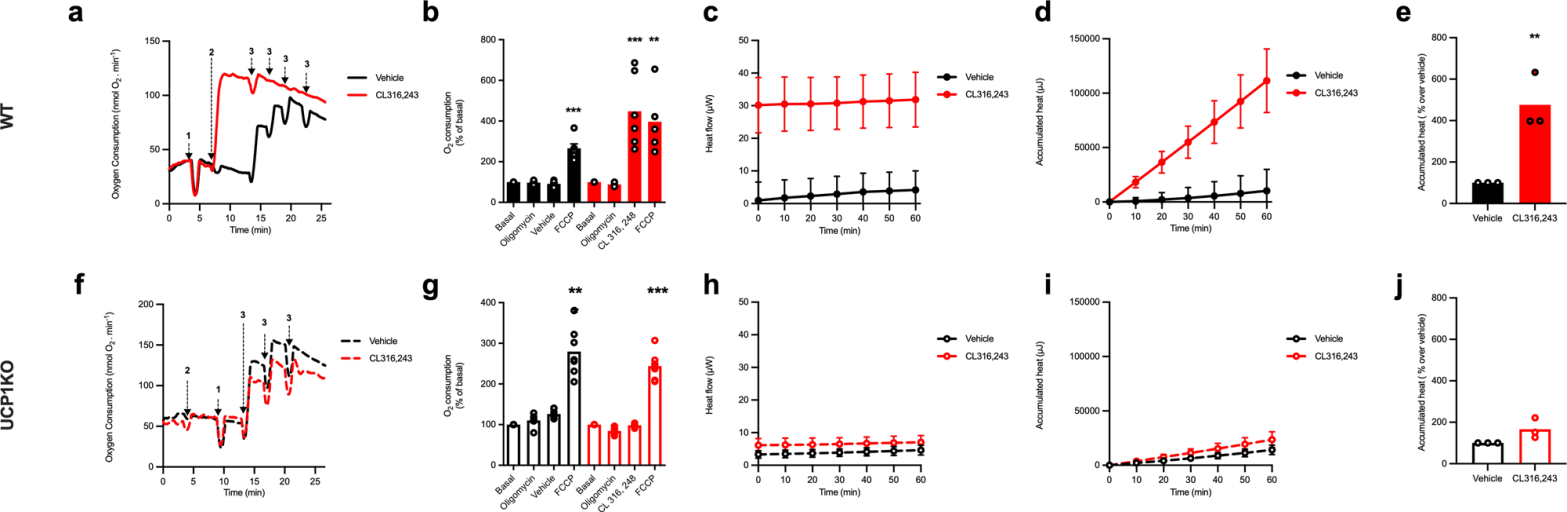
Brown adipocytes as a classical model to demonstrate thermogenesis. **a**, **f** Representative traces of O_2_ consumption over time in exvivo brown adipocytes from WT (solid line) and UCP1 KO mice (dashed line) on a C57BL6 genetic background measured with Oroboros Oxygraph-2K; stimulations are indicated on the graphs, “1” representing addition of oligomycin (2 µg/ml), “2” indicating the addition of Vehicle (black) or 1 µM CL316243 (red), and “3” representing FCCP (40 μM) additions. **b**, **g** Quantifications of O_2_ consumption, normalized as percentage of basal respiration. The data is presented as a bar representing the mean value from independent adipocyte isolations represented by symbols in the scatter plot. Vehicle treated cells are shown in black and CL316243 treated cells are shown in red. Filled bars represent WT cells whereas empty bars denote cells derived from UCP1KO. Experiments were performed on three independent cell preparations for WT (*n* = 3) and 6 for KO mice (*n* = 6). Statistics was calculated using one-way ANOVA, followed by a Dunnett’s multiple comparison test. *P* value for WT Basal vs. FCCP is 0.0003 (***); WT Basal vs. CL316243 is 0.0006 (***), WT Basal vs. FCCP is 0.0040 (**), KO Vehicle Basal vs. FCCP is 0.0001 (***) and KO CL316243 Basal vs. FCCP is >0.0001 (****). Isothermal microcalorimetric measurements of **c**, **h** heat flow, **d**, **i** accumulated heat and **e**, **j** accumulated heat expressed as a percentage over basal for the first hour of the experiment for isolated mature brown adipocytes from UCP1 WT (filled line and bar) and KO (dashed line and empty bar) treated with vehicle (black) or 1 µM CL316243 (red) (*n* = 3). Statistics were calculated using paired *t*-test on the underlying raw data, *P* value for WT Vehicle vs. CL316243 = 0.0089 (**). The curves show mean value $\pm$ SEM. The bars presented in this figure represent the mean value whereas the symbols represent each individual adipocyte isolation.

To assess whether isothermal microcalorimetry could be used for measuring thermogenesis in mature brown adipocytes and to address whether this was UCP1 dependent response, both respirometry and microcalorimetry were performed in mature brown adipocytes isolated from Wildtype (WT) and UCP1 KO mice on the C57BL6 genetic background. We first verified the quality of the isolation by measuring the oxygen consumption of these cells using Oroboros Oxygraph-2K (O2k Oroboros Instruments). Representative traces are shown for brown adipocytes isolated from wildtype (Fig. 3a) and UCP1 knockout mice (Fig. 3f). Addition of oligomycin to inhibit FoF1-ATP-synthase-linked respiration did not induce any significant change in oxygen consumption in brown adipocytes from both wildtype and UCP1 knockout adipocytes.

These cells were then treated with CL316243, which increased the oxygen consumption by approximately 3-fold over basal in WT brown adipocytes (Fig. 3b). Addition of FCCP showed no further increase in oxygen consumption highlighting that brown adipocytes from C57BL6 mice were at maximal oxidative capacity following CL316243 treatment.

As UCP1 independent mechanisms of thermogenesis have been reported to be dependent on ATP synthase activity (13), in contrast to wild type cells, brown adipocytes from UCP1 knockout mice were stimulated with CL316243 in the absence of oligomycin. In these cells (Fig. 3g), CL316243 treatment failed to significantly increase oxygen consumption even in the absence of oligomycin contrasting to previous reports^13^.

This difference could be explained by a difference in ATP metabolism between primary cell culture and primary mature adipocytes and highlighted the importance of development of reliable system for analysis of floating mature adipocytes. Additionally, the higher FCCP response in CL316243-treated WT brown adipocytes is indicative of fatty acid oxidation^17^.

After verifying the quality of adipocyte isolation using respirometry, brown adipocytes from WT and UCP1 KO mice were plated in equal volumes in the wells of the isothermal microcalorimeter. Upon the addition of CL316243 there was a pronounced, significant increase in heat production in mature brown adipocytes isolated from WT mice (Fig. 3c–e). As predicted by data generated using respirometry, CL316243 addition to brown adipocytes form UCP1 KO mice did not significantly induce heat production (Fig. 3h–j).

### Adrenergic stimulation results in concentration dependent heat production in inguinal white adipocytes

Because brite adipocytes normally have a much lower content of UCP1 compared to brown adipocytes, the measurement of brite adipocyte thermogenesis therefore poses substantial challenges (Fig. S1). We thus examined whether isothermal microcalorimetry would be sensitive enough to measure adipocytes with low UCP1 expression. It is known that UCP1 is preserved in ingWAT from new-born mice up-to 3–4 weeks of age, but there exist strain specific differences in the protein content of UCP1 but these have not been studied in inguinal white adipocytes isolated from NMRI mice^18^. Herein, we studied this model in greater detail to establish whether the UCP1 contained within the adipocytes from these mice conferred functional thermogenic capacity. Mature inguinal adipocytes were isolated from young (3 weeks old) NMRI mice and the UCP1 presence was confirmed. As in the case for brown adipocytes, we established the quality of isolation by measuring oxygen consumption using Oroboros Oxygraph-2K. Thereafter, we performed isothermal microcalorimetry in similar preparations of brite adipocytes to validate the thermogenic competence of these cells.

**Fig. 4:**
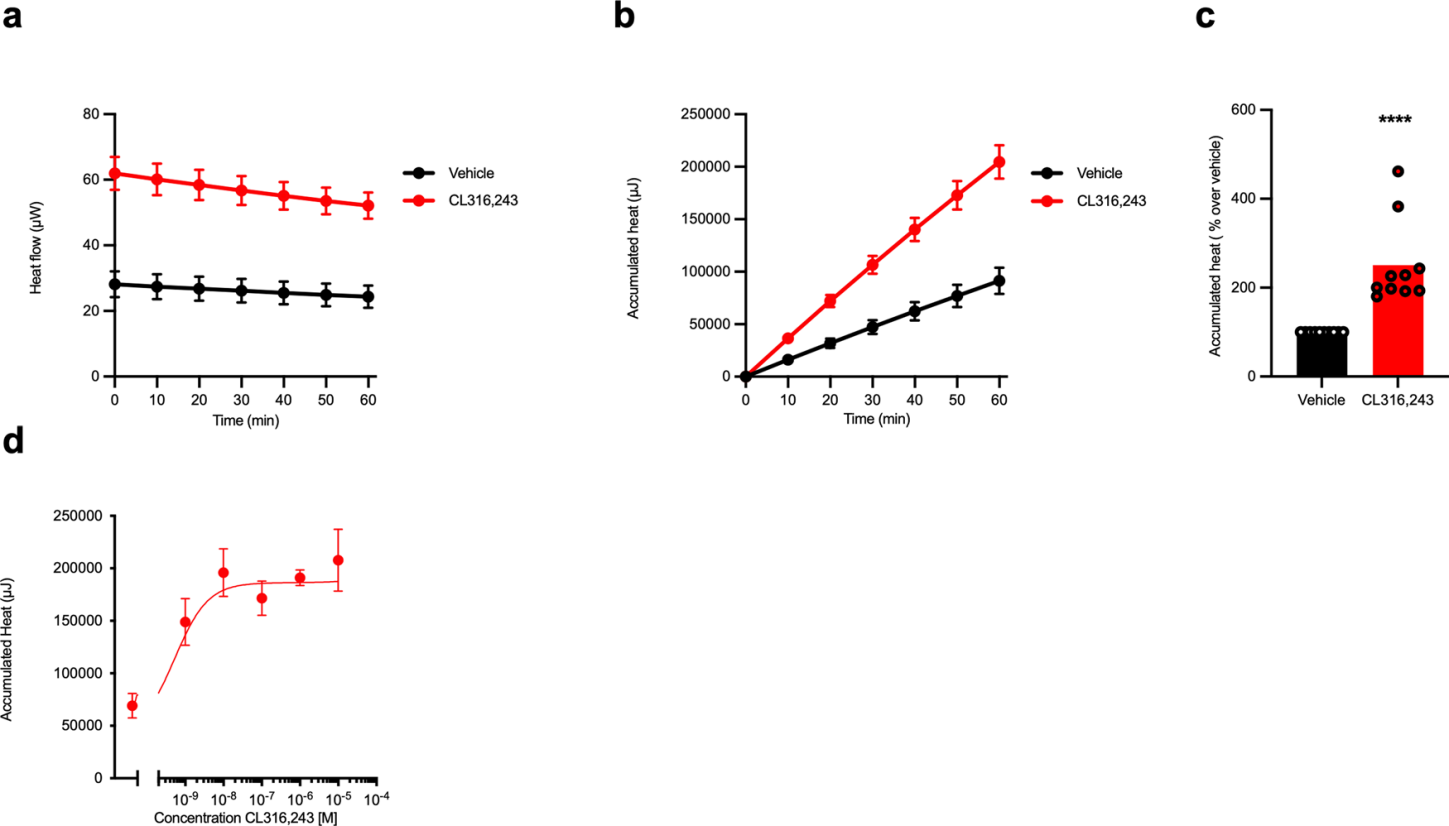
Adrenergic stimulation results in concentration-dependent heat production in inguinal white adipocytes. **a** Heat flow, **b** accumulated heat, and **c** total accumulated heat for the first hour of the experiment for isolated mature inguinal white adipocytes (*n* = 10) from NMRI mice treated with Vehicle (black line and black bar) or 1 µM CL316243 (red line and red bar). **d** Concentration-dependent effects of CL316243 treatment in isolated mature inguinal white adipocytes (*n* = 3), the curve was fit using non-linear regression. Statistics was calculated using paired *t*-test on the underlying raw data. *P* value for vehicle vs. CL316243 is <0.0001 (****). The curves show mean value $\pm$ SEM. The bars presented in this figure represent the mean value whereas the symbols represent each individual adipocyte isolation.

Mature inguinal adipocytes from young (3 weeks old) mice were treated with vehicle or 1 µM of the β_3_-adrenoceptor selective agonist CL316243 in three replicates directly in the wells of the microcalorimeter. There was approximately a two-fold increase in the cumulative heat production in CL316243 treated wells compared to the matched controls showing β_3_-activation in isolated adipocytes to results in increased heat production (Fig. 4a–c). The cumulative heat production in these adipocytes increased in a concentration dependant manner (Fig. 4d), with maximal stimulated heat production at a concentration of 10 nM CL316243. Our results show that isolated mature inguinal white adipocytes from 3-week-old NMRI mice produce quantifiable amounts of heat when stimulated adrenergically even with relatively low amounts of UCP1 (Fig. S1).

### Heat production of mature inguinal white adipocytes is directly correlated to oxygen consumption

Inguinal white adipocytes can under certain conditions develop thermogenic competence^3,4^, i.e., express UCP1. UCP1 content and mitochondrial oxidative capacity are, however, low in these cells. Despite the high total mass of these cells their UCP1-dependent thermogenic potential is limited, as compared to that of brown adipocytes^3^^,6–8^ By being able to use the microcalorimetry and the respirometry in parallel, we could perform parallel measurements of heat production and oxygen consumption in the same cell population.

**Fig. 5:**
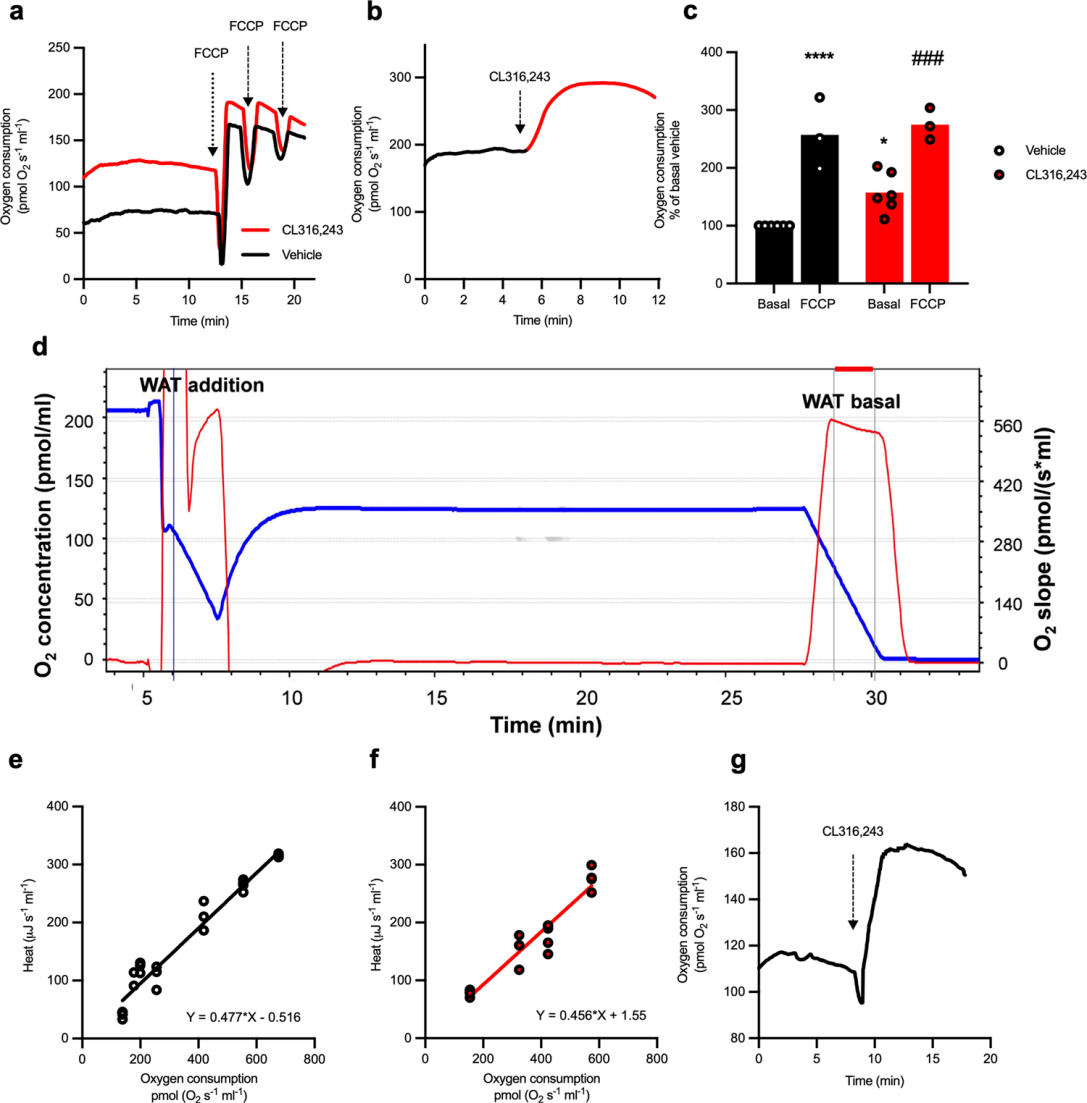
Oxygen consumption of mature inguinal white adipocytes isolated from 3-weeks-old NMRI mice directly correlated to heat production. **a** Representative oxygen consumption traces of freshly isolated mature inguinal white adipocytes pre-treated with vehicle (black line) or CL316243 (red line) and measured in parallel. FCCP was titrated up to 40 µM, additions are indicated as arrows. **b** In another preparation of adipocytes, CL316243 was added to respiring cells during oxygen consumption recording, red indicating oxygen consumption after CL316243 addition. **c** Quantification of mature inguinal white adipocytes oxygen consumption measured as in **a**. The basal oxygen consumption of vehicle-treated cells (black) or oxygen consumption before CL316243 addition (red) was taken as 100%. FCCP-induced or CL316243-induced respiratory rate of the same population cells as well as respiratory rates of measured in parallel CL316243-treated cells in basal state and after FCCP were expressed relatively to this 100%. Data are represented as mean $\pm \,$ SEM, *n* = 3 for FCCP effect and *n* = 6 for CL316243 effect. Statistics was performed using two-way ANOVA followed by a Tukey’s multiple comparison test. *P* value for vehicle basal vs. CL316243 is 0.0399 (*), vehicle basal vs. FCCP is <0.0001 (****) and basal CL316243 vs. FCCP is 0.009 (###). **d** Representative recordings of oxygen concentration (blue line) and oxygen consumption rate (red line) of mature inguinal white adipocytes in Oroboros Oxygraph-2K in parallel to recordings of heat production in isothermal microcalorimeter. To mimic condition of open system in microcalorimeter, an oxygenation was constantly and continuously maintained in Oroboros chamber with exception of several first and last minutes of recordings when chamber was closed. The last period indicated as “WAT basal” corresponds to stable state of heat production (as shown on Fig. 2a). **e** Correlation between oxygen consumption rate (“WAT basal” as shown in **d**) and heat production (measured principally as shown in Fig. 2a simultaneously in 3–4 wells) in vehicle-treated cells. Linear curve fitting (solid line) gives the correlation coefficient equal 0.477. No extra heat production independent on oxygen was observed (heat equal –0.55 when oxygen equal 0). **f** Correlation between oxygen consumption rate and heat production in CL316243-treated cells. Linear curve fitting gives the correlation coefficient equal 0.456 (solid red line). Only very small extra heat production independent on oxygen was observed (heat equal 1.55 when oxygen equal 0). **g** Representative oxygen consumption trace of mature inguinal white adipocytes collected after 40 min measurement in isothermal microcalorimeter stimulated with CL316243 (1 µM). The bars presented in this figure represent the mean value whereas the symbols represent each individual adipocyte isolation.

We first characterized the oxidative properties of the cells. Mature inguinal white adipocytes isolated from young (3 weeks old) NMRI mice preparations were analyzed in the Oroboros Oxygraph-2K. An experiment is shown in Fig. 5a. Untreated white adipocytes had a stable rate of oxygen consumption. These cells responded to addition of the uncoupler FCCP with 2.5-fold increase in oxygen consumption. The ability of these cells to respond oxidatively to FCCP is principally due to the presence of pyruvate in the medium; we have earlier shown that exogenous pyruvate can be oxidized in adipocytes^19^. Treatment of the adipocytes with CL316243 lead to *a* ≈ 50% increase in oxygen consumption (Fig. 5a), suggesting the presence of a small amount of UCP1 in these cells. This limited response to CL316243 was not due to a limitation in mitochondrial respiratory capacity, as adipocytes treated with CL316243 responded to FCCP with an additional increase in oxygen consumption (Fig. 5a–c). Thus, CL316243-induced oxygen consumption was likely the maximal that could be caused by UCP1 activity. This is notable specifically in contrast to brown adipocytes, where CL316243 effect was on maximal level of oxidative capacity (Fig. 5c).

After establishing the oxidative properties of the inguinal white adipocytes, we performed parallel recordings of oxygen consumption and heat production (equal volumes of adipocyte suspensions from the each were added to the Oxygraph and isothermal microcalorimeter, and subsequently the resulting oxygen consumption and heat production measured in parallel). The conditions of the measurements (access to air, stirring, temperature, duration etc.) were made as similar as possible. The oxygen consumption measurements are exemplified in Fig. 5d. The white adipocyte suspension was added to the Oxygraph and the lid replaced. This led to a rapid decrease in oxygen tension (blue line) due to the high rate of oxygen consumption (red line). As the isothermal microcalorimeter allows for constant access to oxygen (air) during the time period measured, we paralleled this condition by removing the lid; this led to rapid increase in oxygen tension (and thus to an apparent lack of oxygen consumption). When the white adipocytes had been incubated for the same time as the routine calorimetric experiments (25–30 min), the lid was replaced for about 2 min and the rate of oxygen consumption recording during this time. Note that the rate was the same as the initial rate; the long incubation did thus not negatively affect the cells (Fig. 5g). In addition, it is important to consider that equal volumes of adipocyte suspension from the same preparation were used to measure heat and oxygen consumption in microcalorimeter and Oroboros Oxygraph O2K, respectively.

When these parallel experiments had been performed in a series of preparations of inguinal white adipocytes, we could examine the relationship between oxygen consumption and heat production in these cells. According to our analysis, oxygen consumption and heat production are directly proportional (Fig. 5e, f). Linear fitting of relation between oxygen consumption rate and heat production in unstimulated inguinal adipocytes yielded a coefficient of 0.477 µJ per pmol O_2_ (Fig. 5e). The expected heat (energy) equivalent for a given amount of oxygen consumed (oxycaloric equivalent) depends on the type of substrate burned. According to Elliot & Davison^20^ for carbohydrate, the expected value is 0.472 µJ heat produced per pmol O_2_ and for fat it is 0.439 µJ per pmol O_2_. Thus, in our hands, inguinal adipocytes in media supplemented with pyruvate burned carbohydrate—as expected^19^. Treatment of cells with CL316243 slightly changed the obtained oxycaloric equivalent to 0.456 µJ per pmol O_2_ (Fig. 5f), indicating that—in addition to carbohydrate—lipid-derived substrates were used for oxidation in adipocytes under β_3_-adrenergic stimulation.

Notable, as the observed calorific equivalent did not exceed the expected, no extra heat production independent on oxygen consumption was observed, these results do not support the existence of any alternative mechanism of heat production, independent of oxygen consumption.

To examine whether there are negative effects of the 30 min incubation in the isothermal microcalorimeter on the mitochondrial function of white adipocytes, we analyzed the effects of mitochondrial agents as well as CL316243 on oxygen consumption of cells collected from after the 30 min of calorimetric measurements (Fig. 5g). Addition of CL316243 induced oxygen consumption similarly as in freshly isolated cells. Thus, the 30 min incubation (maintenance) of cells inside isothermal microcalorimeter did not have any detrimental effect on adipocyte function.

### UCP1 is the primary driver of thermogenesis in brite adipocytes

**Fig. 6:**
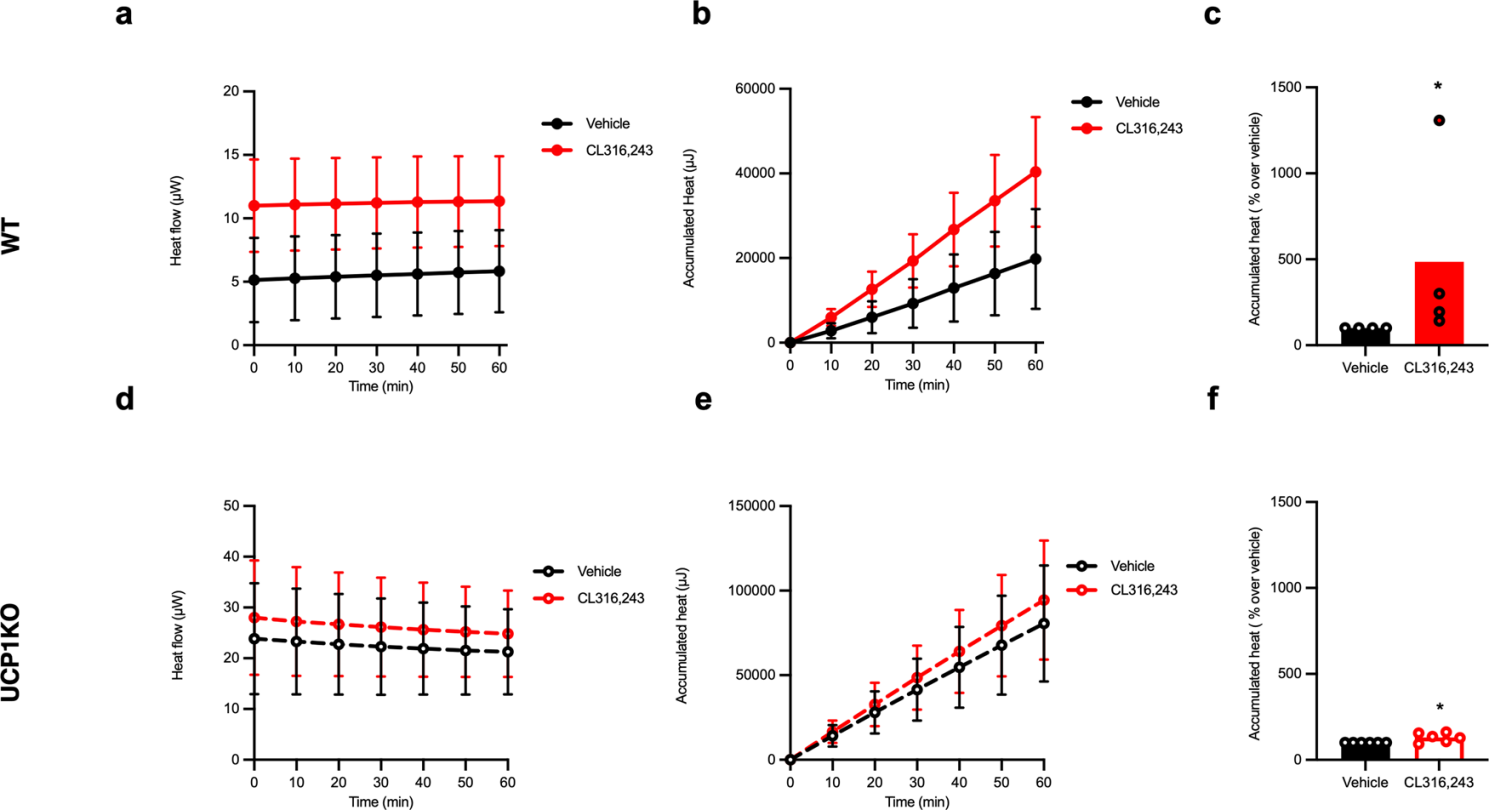
UCP1 is the primary driver of thermogenesis in brite adipocytes. **a**, **d** Heat flow, **b**, **e** accumulated heat, and **c**, **f** accumulated heat expressed as a percentage increase over basal for the first hour of the experiment for isolated mature inguinal from WT mice (*n* = 4, solid line and solid bar) and UCP1 KO mice (*n* = 6, dashed line and empty bar) on the same genetic background treated with either vehicle (black) or 1 µM CL316243 (red). Statistics was calculated using paired *t*-test on the underlying raw data. *P* value for Vehicle vs. CL316243 is 0.0114 (*) in WT and 0.0172 (*) in UCP1 KO cells. The curves show mean value ± SEM. The bars presented in this figure represent the mean value whereas the symbols represent each individual adipocyte isolation.

As UCP1-independent mechanisms are purported to contribute to heat production in brite adipocytes, we measured adrenergic heat production induced in mature inguinal white adipocytes freshly isolated from WT (Fig. 6a–c) and UCP1 KO (Fig. 6d–f) mice. CL316243 stimulation increased heat production in adipocytes independently of the presence of UCP1. Of note is the fact that the fold change over basal was considerably lower in UCP1 KO adipocytes suggesting that the absence of UCP1 blunts adrenergic heat production in these cells; the modest increase in heat production could be a consequence of increased substrate oxidation as nonspecific protonophoric effects of FFAs were eliminated in the experimental design due to the presence of BSA in the stimulation medium^21,22^. Additionally, our data suggests that isothermal microcalorimetry is a sensitive method to screen for both UCP1-dependent and independent mechanisms of heat production in brite adipocytes as very modest increases in heat production can be discriminated with a high degree of reproducibility.

### hMADS retain the capacity to produce heat in vitro

**Fig. 7:**
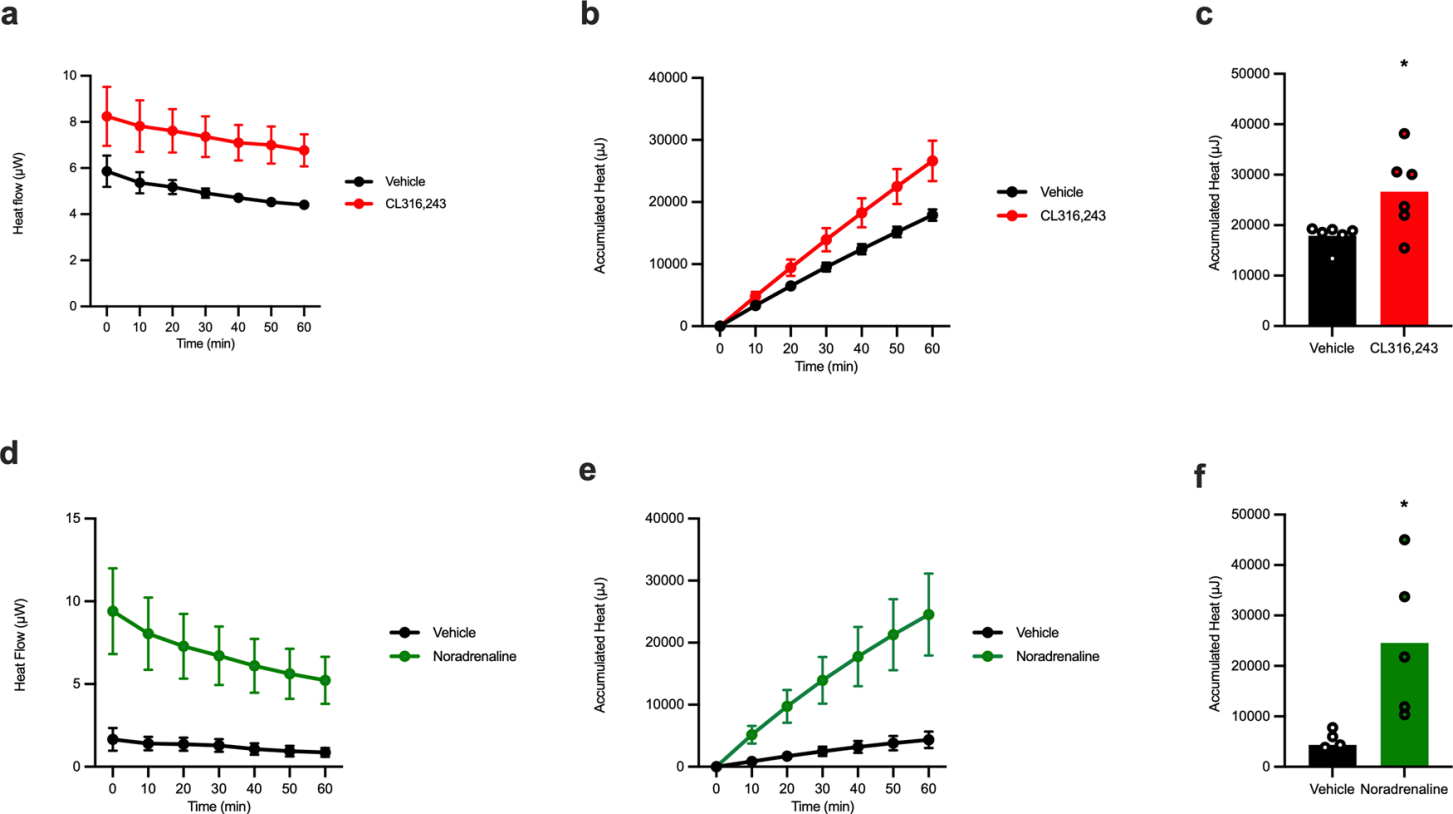
hMADS retain the capacity to produce heat in vitro. **a**, **d** Heat flow, **b**, **e** accumulated heat, and **c**, **f** total accumulated heat for the first hour of the experiment for differentiated Human multipotent adipose-derived stem cells (hMADS) treated with either Vehicle (black line and black bar), CL316243 (*n* = 6, red line and red bar) and Noradrenaline (*n* = 5, green line and green bar) plated in separate wells. Statistics was calculated using *t*-test. *P* value for Vehicle vs. CL316243 is 0.0265 (*) and Vehicle vs. Noradrenaline is 0.0172 (*). The curves show mean value $\pm$ SEM. The bars presented in this figure represent the mean value whereas the symbols represent each individual well containing independent preparations of hMADS.

There are marked differences in murine and human thermogenic adipocytes, both in terms of the heterogeneity they present and more recently also the predominant adrenergic receptor that is driving heat production in these cells^23^. It is therefore important to measure efficacy of novel sympathomimetics in human thermogenic adipocytes. To demonstrate the sensitivity of isothermal microcalorimetry for measuring heat production in human cells, we differentiated human multipotent adipose-derived stem cells (hMADS) into functional brown-like adipocytes as published in individual wells of the microcalorimeter^24^. Stimulation with 1 µM of CL316243 resulted in increased heat production (Fig. 7a–c). Upon treatment with noradrenaline, the hMADS responded similarly (Fig. 7d–f); compared to CL316243 the increase in heat production over basal was much higher. Our data demonstrates that hMADS produce heat upon adrenergic stimulation and therefore represent a functionally thermogenic cell line.

## Discussion

Calorimetry has been used in the past to measure heat production in isolated brown adipocytes^15,16^. However, traditional isothermal calorimetric approaches have suffered from low sensitivity, the requirement for a large amount of sample material and the inability to perform the assay in sufficient throughput. Furthermore, advances in computational power have allowed modern isothermal microcalorimeters to better resolve power time curves improving both sensitivity and resolution. Not only is isothermal microcalorimetry a powerful tool for the screening of therapeutic compounds and lead compound selection, in this case with the capacity to activate brite thermogenesis, it is also sensitive enough to measure the very low thermogenic output of brite adipocytes allowing for the measurement of concentration-dependent increases in heat production.

The data is presented in a real-time format, where the rate of heat flow, measured in µW is plotted against time. This data can be easily transformed into accumulated heat. The large cell/media as well as media/gas phase ratios of the 48-well sized wells allows for long term measurement of heat production making this technique optimal for the study of chronic effects of various agonists on thermogenesis in brown and brite adipocytes. In addition, a key advantage of this technique over other comparable methods is the ability to measure heat production in floating cells. Furthermore, these are not subjected to constant stirring and the associated shear forces thereby potentially limiting artefacts when recording heat production. The media/gas ratio within the wells can also be modified to theoretically measure both oxygen dependent and independent heat production. As demonstrated, the cells subjected to isothermal microcalorimetry remain viable during the length of the experimental period due to the non-destructive nature of the instrumentation, making it ideal for use in longer term experiments. Furthermore, the protocols described here in can be easily modified to accommodate for measuring heat in other mammalian cells for a variety of diverse applications. There are however some limitations to the technique, the equilibration phase of the instrument coupled with a lack of microinjection system makes it difficult to measure acute thermogenic responses. Additionally, to enable the maximum accuracy the instrument needs to be maintained in a temperature controlled environment.

In isolated brown adipocytes, we have shown that the addition of CL316243 resulted in a pronounced, several fold increase in heat production presumably due to the activation of UCP1. As isothermal microcalorimetry allows for the measurement of heat in the low microwatt range, we hypothesized that this instrumentation could be used for the measurement of heat production in brite adipocytes isolated from inguinal white adipose tissue from young mice. Though UCP1 is present in these adipocytes, in comparison to brown adipocytes, it is in much lower amounts and is susceptible to genetic variability among mouse strains^18^. The presence and the changes in UCP1 amount have been well documented in C57BL6 and A/J strains. However, the thermogenic capacity and the competency of mature brite adipocytes from the strain used in present experiments has not been characterized. Stimulation with β_3_-adrenoceptor agonist CL316243 resulted in significantly higher heat production in isolated mature brite adipocytes; this is in accordance with increased rates of oxygen consumption in these cells when treated with CL316243. These findings are noteworthy as we have established that brite adipocytes isolated from 3-week-old NMRI mice are thermogenically competent and together with isothermal microcalorimetry, can be used as a model for measuring brite adipocyte thermogenesis.

We ran a number of experiments to ensure that the responses observed varied linearly with the number of cells present within the wells. This was indeed the case in both stimulated and unstimulated brite adipocytes and the effects were easily reproducible. This highlights the dynamic range of the instrument and its ability to measure minute amounts of heat production in brite adipocytes. In addition to UCP1 mediated thermogenesis, alternative, UCP1-independent pathways have been shown to contribute to heat production in brite adipocytes^6–8^. The existence of UCP1-independent pathways suggests that estimation of heat production based on oxygen consumption as a proxy measurement might be underestimated, making isothermal microcalorimetry an optimal system for the measurement of UCP1-independent thermogenic activation in brite adipocytes. Upon further validation of the instrumentation by comparing it to respirometry, we observed a linear relationship between the heat production and oxygen consumption expressed as the oxy-caloric equivalent in mature inguinal white adipocytes that had been isolated from the same preparations. This relationship was not observed to exceed the oxy-caloric equivalent highlighting that alternative oxygen independent mechanisms do not significantly contribute to heat production in inguinal white adipocytes at least in a stimulated ex-vivo state. The calorimetric to respirometry ratio has been calculated for a variety of cells including rat brown adipose tissue^25,26^. A negative shift in this ratio represents either uncoupling or enhanced substrate cycling^27^. Isolated hamster brown adipocytes present an average calorimetric to respirometric ratio of 490 kJ/mol of oxygen consumed^16,28^ representing a fully aerobic metabolism. Our experimentally determined value of this ratio in brite adipocytes falls within a similar range highlighting little contribution of anaerobic catabolism towards the observed heat production.

In addition, the ratio between the rate of oxygen consumption and heat production can provide valuable insights into the substrate preference of these cells when subjected to pharmacological agonists. The substrate fluxes in brown adipocytes upon isoproterenol stimulation have been a subject of an in-depth study recently^13^. As expected, isoproterenol greatly induced FFA production a fraction of which were reported to be beta oxidized. The shift in the observed in oxy-caloric ratio in brite adipocytes upon stimulation with CL316243 seems to mirror the aforementioned observations in brown adipocytes, signifying an increased usage of lipid derived substrates.

Collectively, our data suggests that the method developed herein, for the measurement of oxygen consumption and heat production in parallel, is a powerful tool for the study oxygen and UCP1 independent mechanisms of thermogenesis in brite adipocytes. In addition, the calorimetric to respirometric ratio determined in our study does not allude to the presence of any anaerobic mechanisms of heat production in intact brite adipocytes in basal or stimulated conditions.

Our results imply that the presence of UCP1 is fundamental for β_3_-adrenoceptor mediated oxygen consumption and the resulting heat production in mature brown adipocytes, in agreement with earlier data^14^. Mitochondrial defects and a lack of a capacity to utilize pyruvate in brown adipocytes from UCP1 knockout mice could in part contribute to the absence of a stimulated response in these cells^29^. However, in our present experiments we observe no differences in the FCCP-induced oxygen consumption between WT and UCP1KO brown adipocytes showing that the mitochondria are indeed functional (Fig. S2). Downregulation of the adrenoreceptor and the lipolytic machinery has been shown in UCP1 knockout brown adipocytes^30^ isolated from old mice and on HFD; in contrast it has earlier been shown that lipolysis is not affected in preparations similar to those used here^14^. Thus, this effect is primarily age and diet dependent; at 3 months old the β_3_-AR together with other genes associated with fatty acid metabolism genes are reported to be higher in brown adipose tissue in UCP1KO mice. Since the animals used in the present study are approximately of this age, one would expect the adrenergic machinery to be intact and therefore not be causative of the phenotypic observations in UCP1KO brown adipocytes.

This lack of a stimulated response is in stark contrast to cultured brown adipocytes growing in the presence of differentiating agents^13^, where adrenergic stimulation results in an increased ATPase dependent increases in oxygen consumption even in cells derived from UCP1 KO mice. These divergent responses allude to marked molecular and functional differences in freshly isolated adipocytes in comparison with those grown in culture, thereby highlighting the necessity for the development of systems that can allow for direct bioenergetic analysis in mature cells, which more accurately reflect their inherent physiologic roles. Additionally, the induction of heat production over basal was much higher in brown adipocytes as compared to brite adipocytes potentially due to the increased amount of UCP1 protein in the former signifying the importance of UCP1 content in potentiating adrenoceptor mediated thermogenic responses in these cells.

Notably, it is important to highlight a small, yet significant increase in adrenergic induced heat production in inguinal white adipocytes isolated from UCP1 KO mice. As there were sufficient quantities of BSA present in the medium for adequate inhibition of non-specific FFA mediated protonophoric interactions^21^, the increase in thermogenic activity in these cells can be attributed to increased substrate oxidation. However, this induction of heat production was marginal in comparison to that observed in brite adipocytes from WT C57BL6 animals effectively showing that in the absence of UCP1, while adrenergic stimulation does indeed result in heat production, alternative mechanisms contributing to this effect are not induced in sufficient quantities to fully compensate for the lack of UCP1^8^.

As to whether the technology can potentially be used for human tissues, we show that this method is sensitive enough to measure the relatively low production of heat in cultured hMADS differentiated to acquire a more “brite-like” phenotype. It has been established that these cells express the β_3_-adrenoeceptor and respond to both noradrenaline and CL316243 stimulation^24^. Compared to the cells derived from mice, adrenergic stimulation resulted in a much lower thermogenic response in hMADS potentially due to much lower expression of UCP1. While both noradrenaline and CL316243 were able to induce significant heat production in hMADS, there is an important need for testing the thermogenic response induced using different agonists to identify more potent stimulators of heat production. In contrast to rodent models, brown/brite adipose tissue in humans is relatively heterogeneous^31^, it is therefore essential to account for these differences when designing therapeutics aimed at the activation of thermogenesis in humans.

Furthermore, recent advances showing that thermogenesis in humans is mediated primarily through the β_2_-adrenoceptor^23^ highlight the need to develop assays wherein human adipocytes can be grown, and used to screen for compounds that can activate thermogenesis in these cells, both UCP1-dependent and UCP1-independent. The technology described in this paper could be an effective tool for this purpose, as the efficacy of various treatments on thermogenesis can be tested in vitro, including on biopsies from patients.

In conclusion, we have presented here a method for assaying thermogenic activity in murine and human adipocytes that offers considerable advantages when compared to more conventional respirometric approaches allowing for the use of floating cells and a higher throughput. Using direct calorimetric measurements of heat production, we have established the fundamental importance of UCP1 in catalysing adrenergic induced thermogenic responses in isolated brite adipocytes ex vivo.

## Methods

### Isothermal microcalorimeter assay

For all experiments, we used the calScreener™ a 48-channel isothermal microcalorimeter (Symcel SverigeAB, Spånga, Sweden), with its corresponding 48-well plate (calPlate™) as previously described. Each well consists of a screw-capped titanium vial, with sterile single use plastic insert of cell-culture and microscopy grade quality, in which a maximum of 300 μl media can be added (total volume of insert is 600 µl). Data was continuously collected with the corresponding calView™ software (Version 1.0.33.0, 2016, Symcel Sverige AB). For the assays, the machine was set and calibrated at 37 °C. General handling and device manipulation were done according to the manufacturer’s recommendations. The configuration of the system is based on a twin calorimeter setup with 16 reference positions connected to two sample positions each, making a total of 32 positions available for biological samples. The reference positions are used to correct for environmental factors such as external temperature changes, improving the sensitivity of the system.

For sample loading the mature adipocyte cell suspension was gently transferred into 30-well inserts of the plate in a volume of 200 µl/well. The other 2-well inserts were used as negative controls and loaded with only media to measure and exclude background heating or possible heat reactions of the inserts or working media. The inserts where transferred into the calPlate^TM^, and the stimulation or control was added to the wells (1 nM–100 µM). On a 37 °C heat bock, each vial in the plate was sealed using a specific torque. The calPlate^TM^ is inserted into the first of two temperature equilibrium zones of the machine for 10 min, and then incubated in the second zone for an additional 20 min before entering the measurement position.

Insertion of the sample plate into the measurement position creates thermal anomalies due to friction and pressure effects on the sensors used in isothermal calorimetry. The total equilibrium time is 30 min for pre-equilibrium followed by a further 30 min stabilization time after final sample positioning. The first viable data will be recorded 60 min after the initial sample loading. Therefore, it is essential that all the measurements are carried out with a controlled starting time. Furthermore, baseline measurements are taken for each sample position at the lowest point of the curve, where either the cells are dead or the substrates to provide energy and oxygen has run out. Internal baseline adjustment for every position is essential to achieve correct heat flow measurement data. Each well is set to its baseline for comparison between samples and between consecutive runs. The baseline value was routinely monitored to account for any drifts this was on average less than 0.2 µW.

### Isolation of mature brown adipocytes

All experiments were approved by the Animal Ethics Committee of the North Stockholm region (DNR 15083-2020). Mature brown adipocytes were isolated by collagenase digestion of pooled BAT depots (interscapular, axillary and cervical). On each experiment, three male C57BL/6 or UCP1 KO mice (12-week-old) were killed by CO_2_ anesthesia and decapitated. BAT depots were put into Krebs Ringer phosphate buffer (1.4 mM KH_2_PO_4_, 3.76 mM NaH_2_PO_4_, 16.74 mM Na_2_HPO_4_, 110.38 mM NaCl, 5.55 mM KCl, 1.4 mM MgSO_4_, 1.5 mM CaCl_2_, 10 mM glucose, 10 mM fructose, 4% fatty acid-free BSA, pH 7.4), thereafter referred as KRPB.

Tissue was minced with scissor and incubated in KRPB 1.3 mg/ml collagenase type I (Sigma-Aldrich) in a shaking water bath (100 rpm, 37 °C), vortexing every 2 min. After 7 min, the buffer was discarded through filtration, the tissue was minced again and incubated in KRPB 0.67 mg/ml collagenase type I for 30–45 min in a shaking water bath (100 rpm, 37 °C), vortexing every 5 min. The buffer, containing the adipocytes, was filtered through a 250 μm nylon filter, the filtrate centrifuged for 10 min at 2 × *g*. The infranatant was discarded and KRPB was added to the adipocyte suspension. The remaining tissue was incubated another 20–30 min in KRPB 0.33 mg/ml collagenase type I, vortexing every 5 min and filtered. The filtrate was centrifuged 10 min at 2 × *g*, and the adipocytes were collected. The two adipocyte suspensions were pooled and washed by floating for 1 h.

### Isolation of mature white/brite adipocytes

All experiments were approved by the Animal Ethics Committee of the North Stockholm region (DNR 15083-2020). For tissue isolation, 3–4 weeks old male Naval Medical Research Institute (NMRI), C57BL/6 or UCP1 KO (B&K, Stockholm, Sweden), mice were kept at room temperature provided with food and water ad libitum, with a 12:12-h light-dark cycle. Animals were euthanized with CO_2_ followed by cervical dislocation. Thereafter, inguinal white adipose tissue (IngWAT) depots were isolated from six mice (as previously described (ref.)), pooled and finely cut with a scissors in high glucose DMEM and transferred to × volume of digestion solution 0.2% (wt/vol) collagenase (type II; Sigma) in a buffer consisting of 0.1 M HEPES (pH 7.4) 123 mM NaCl, 5 mM KCL, 1 mM CaCl_2_, 4.5 mM glucose, and 1.5% (wt/vol) BSA. The digestion was performed for 30 min, shaking at 37 °C. The subsequent cell suspension was filtered through a 250-µm pore-size nylon filter (Sintab, Oxie, Sweden), and aspirated with a 10 ml syringe to generate a homogenous single-cell suspension. In order to enrich for the mature adipocytes and inactivate collagenase, the filtered cell suspension in the syringe was vertically placed on ice for 20 min. The stromal vascular fraction at the bottom of the syringe and the buffer was gently pushed out, and the remaining floating mature adipocyte cells were mixed with working media consisting of high glucose DMEM, 4% (wt/vol) Fraction V fatty acid free BSA, 10 mM HEPES, 50 U/ml penicillin, and 50 µg/ml streptomycin (all from Sigma).

### Mature brown and white adipocyte oxygen consumption

Oxygen consumption of isolated mature brown and inguinal white adipocytes was measured using a high-resolution oxygraph (Oroboros Oxygraph-2K, Austria). Oxygen concentration and rate of oxygen consumption were recorded continuously using DAT LAB software 6. Mature adipocytes derived from male mice from separate preparations were isolated and were resuspended in respiration medium (DMEM 4500 mg/l glucose (Sigma, Germany) to which was added 4% fatty acid-free BSA (Roche, Germany) and 5 mM pyruvate (Sigma, Germany)) that had been pre-heated to 37 °C. 2.1 ml of cell suspension from each distinct adipocyte preparation was added to the oxygen electrode chambers, where it was magnetically stirred, and kept at 37 °C. The chamber was closed with the lid (if not otherwise indicated) and the cells were incubated until a stable basal respiratory rate was reached. After this, additions (oligomycin (2 μg/ml, Sigma-Aldrich), CL316,243 (1 μM, Sigma-Aldrich) and FCCP (titration with 40 μM until maximal oxygen consumption rate was obtained, Sigma-Aldrich) were successively made to the chamber with a Hamilton syringe. For the experiments on parallel measurement of heat production and oxygen consumption, adipocytes were treated with CL316243 (1 μM, Sigma-Aldrich) or vehicle (dd water) just before the start of recordings, and aliquoted into the wells of CalScreener and in Oroboros oxygraph chambers. To mimic the open system conditions of the CalScreener, oxygenation was maintained in the oxygraph chamber; i.e., the lid was not replaced, with the exception of the first several minutes and the last minutes when the recordings were made and the chamber was closed. The oxygen consumption rate values were expressed in pmol O_2_/(sec × ml) or represented as percentage of basal oxygen consumption rates.

### Immunoblotting

A sample from each measurement was collected and mixed 1:1 with SDS buffer, and 6 µl sample was loaded and separated on a 12% SDS-gel. The proteins where transferred onto a PVDF-membrane and before blockage with 5% milk in TBS-T of the membrane, the membrane was stained with ponceau. The membrane was then incubated overnight at 4 °C with the primary antibody (anti-UCP1 made in house, 1:2000) and further incubated with the secondary antibody (anti-rabbit-HRP coupled, 1:2000 CST, in 2% BSA in TBS-T) at room temperature for 1 h. After washing the membrane in TBST, the proteins could be visualized by a CCD-camera using enhanced HRP- solution (Thermo Fisher).

### Human multipotent adipose-derived stem cells (hMADS)

The hMADs were differentiated into functional brown-like adipocytes as in the inserts of the calPlate^TM^ as shown^24^. Undifferentiated cells were grown in Dulbecco’s Modified Eagle’s Medium (DMEM) supplemented with 10% Fetal Bovine Serum (FBS), 2.5 ng/ml of hFGF2 (Peprotech), 100 U/ml penicillin, 100 μg/ml streptomycin, and 10 mM HEPES, in a 37 °C incubator with 5% CO_2_. Upon reaching confluence the hFGF2 was removed from the medium, and differentiation was induced 2 days of post-confluence (Day 0). The cells were maintained in DMEM/Hams F12 medium with 500 μM of isobutyl methylxanthine (IBMX), 1 μM of Dexamethasone, 10 nM of insulin, 10 µg/ml of transferrin, 0.2 nM triiodothyronine and 100 U/ml penicillin, 100 μg/ml streptomycin, and 10 mM HEPES. IBMX and Dexamethasone were removed from the medium at day 2. The cells were supplemented with rosiglitazone from days 2 to 9 and 14 to 18. After 3 weeks of differentiation, the inserts were transferred to the calPlate^TM^. Cells were stimulated with the β_3_ agonist CL316243 or corresponding control after transferring the inserts to the calPlate^TM^ and subsequently the the calScreener^TM^.

### The calView^TM^ program

The calView software is a proprietary software for the data collection and analysis of calScreener IMC data. The direct measurement in IMC is heat flow (power in J/s) as a function of time. The heatflow gives the kinetic behavior and response of the sample over time. Data can also be expressed as the total accumulated heat (energy expressed in J) over time as an alternative data presentation of the cellular response to treatment. Total energy release can also be plotted as a function of compound concentration for dose-response measurements.

### Statistics and reproducibility

The statistical tests used were computed using Prism 9.0 and are specified in the figure legends. Briefly, two-tailed paired *t*-tests were performed on underlying raw data to compare the stimulatory effect of agonist treatment in murine adipocyte preparations and unpaired *t*-test were performed for hMADS. One-way anova and two way anova were performed to calculate statistics where multiple groups were present. Each data point presented in the figures represents a pooled preparation of iWAT or BAT from 3 to 4 mice, and was considered as one N plated in at least two replicates in the isothermal microcalorimeter. One replicate was considered as one well in the microcalorimeter from the same adipocyte isolation. Each experiment was performed at least three times with at least two replicates per condition in the microwells of the isothermal microcalorimeter.

### Reporting summary

Further information on research design is available in the Nature Research Reporting Summary linked to this article.

## Supplementary information

Supplementary Figures

Description of Supplementary Files

Supplementary Data 1

Supplementary Data 2

Supplementary Data 3

Supplementary Data 4

Supplementary Data 5

Supplementary Data 6

Reporting Summary

## Data Availability

All source data is available upon reasonable request from the corresponding author. The data used to generate the figures is available as Supplementary Data 1–7.
