# Supplementary material for: Isothermal microcalorimetry measures UCP1-mediated thermogenesis in mature brite adipocytes
Source: Commun Biol. 2021 Sep 21;4:1108. doi: 10.1038/s42003-021-02639-4 (PMC8455563; doi:10.1038/s42003-021-02639-4)
Supplement: Supplementary file 1 — Supplementary Figures [file 42003_2021_2639_MOESM1_ESM.pdf]

Supplementary Data

Figure S1 – Immunoblot showing relative UCP1 protein amounts in brown and inguinal white adipocytes

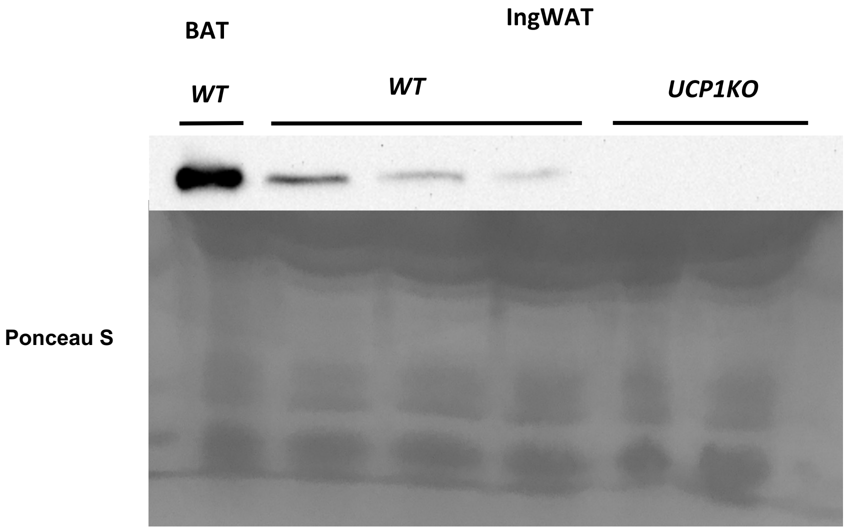

Figure S2 – Δ FCCP response of brown adipocytes from C57BL6 and UCP1KO mice.

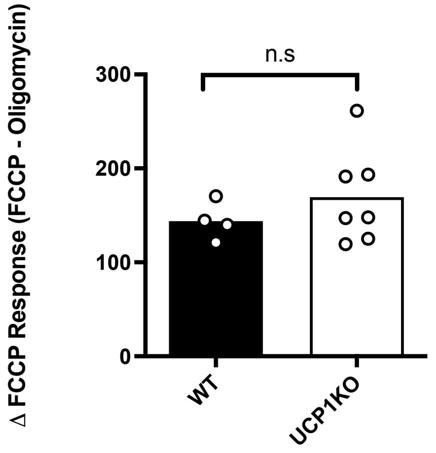

Fig. S2 – Spare respiratory capacity as measured by the FCCP response in brown adipocytes isolated from WT and UCP1KO animals. Each point represents an individual adipocyte isolation.
