# Supplementary material for: Isothermal microcalorimetry measures UCP1-mediated thermogenesis in mature brite adipocytes
Source: Commun Biol. 2021 Sep 21;4:1108. doi: 10.1038/s42003-021-02639-4 (PMC8455563; doi:10.1038/s42003-021-02639-4)
Supplement: Supplementary file 2 — Description of Supplementary Files [file 42003_2021_2639_MOESM2_ESM.pdf]

Description of Additional Supplementary Files

File name: Supplementary Data 1

Description: Source data used to generate Figure 2

File name: Supplementary Data 2

Description: Source data used to generate Figure 4.

File name: Supplementary Data 3

Description: Source data used to generate Figure 4.

File name: Supplementary Data 4

Description: Source data used to generate Figure 5.

File name: Supplementary Data 5

Description: Source data used to generate Figure 6.

File name: Supplementary Data 6

Description: Source data used to generate Figure 7.
