# Supplementary material for: Isothermal microcalorimetry measures UCP1-mediated thermogenesis in mature brite adipocytes
Source: Commun Biol. 2021 Sep 21;4:1108. doi: 10.1038/s42003-021-02639-4 (PMC8455563; doi:10.1038/s42003-021-02639-4)
Supplement: Supplementary file 9 — Reporting Summary [file 42003_2021_2639_MOESM9_ESM.pdf]

nature research

Corresponding author(s): Tore Bengtsson

Last updated by author(s): Aug 11, 2021

# Reporting Summary

Nature Research wishes to improve the reproducibility of the work that we publish. This form provides structure for consistency and transparency in reporting. For further information on Nature Research policies, see our Editorial Policies and the Editorial Policy Checklist.

## Statistics

For all statistical analyses, confirm that the following items are present in the figure legend, table legend, main text, or Methods section.

| n/a | Confirmed | |
|---|---|---|
| ☐ | ☒ | The exact sample size (*n*) for each experimental group/condition, given as a discrete number and unit of measurement |
| ☐ | ☒ | A statement on whether measurements were taken from distinct samples or whether the same sample was measured repeatedly |
| ☐ | ☒ | The statistical test(s) used AND whether they are one- or two-sided<br>*Only common tests should be described solely by name; describe more complex techniques in the Methods section.* |
| ☒ | ☐ | A description of all covariates tested |
| ☐ | ☒ | A description of any assumptions or corrections, such as tests of normality and adjustment for multiple comparisons |
| ☐ | ☒ | A full description of the statistical parameters including central tendency (e.g. means) or other basic estimates (e.g. regression coefficient) AND variation (e.g. standard deviation) or associated estimates of uncertainty (e.g. confidence intervals) |
| ☐ | ☒ | For null hypothesis testing, the test statistic (e.g. *F*, *t*, *r*) with confidence intervals, effect sizes, degrees of freedom and *P* value noted<br>*Give P values as exact values whenever suitable.* |
| ☒ | ☐ | For Bayesian analysis, information on the choice of priors and Markov chain Monte Carlo settings |
| ☒ | ☐ | For hierarchical and complex designs, identification of the appropriate level for tests and full reporting of outcomes |
| ☐ | ☒ | Estimates of effect sizes (e.g. Cohen's *d*, Pearson's *r*), indicating how they were calculated |

*Our web collection on statistics for biologists contains articles on many of the points above.*

## Software and code

Policy information about availability of computer code

| | |
|---|---|
| Data collection | Symcel CalView was used to extract raw data for heat generation. |
| Data analysis | Graphpad Prism 9.0 was used for making graphs, and subsequent data analysis. |

For manuscripts utilizing custom algorithms or software that are central to the research but not yet described in published literature, software must be made available to editors and reviewers. We strongly encourage code deposition in a community repository (e.g. GitHub). See the Nature Research guidelines for submitting code & software for further information.

## Data

Policy information about availability of data

All manuscripts must include a data availability statement. This statement should provide the following information, where applicable:

- Accession codes, unique identifiers, or web links for publicly available datasets
- A list of figures that have associated raw data
- A description of any restrictions on data availability

All raw data is available upon reasonable request

# Field-specific reporting

# Life sciences study design

All studies must disclose on these points even when the disclosure is negative.

| | |
|---|---|
| Sample size | There were no power calculations performed prior to experimentation however n=3 was chosen as the minimum sample size |
| Data exclusions | No data was excluded from analysis. |
| Replication | Attempts at replication were successful. |
| Randomization | The stimulation scheme for adipocyte treatment in the isothermal microcalorimeter was routinely changed so as to discount well specific differences between experimental runs. |
| Blinding | Blinding was not executed as the same researcher isolating adipocytes deriving from the different genotypes was also performing the subsequent calorimetric experiments |

# Reporting for specific materials, systems and methods

We require information from authors about some types of materials, experimental systems and methods used in many studies. Here, indicate whether each material, system or method listed is relevant to your study. If you are not sure if a list item applies to your research, read the appropriate section before selecting a response.

## Materials & experimental systems

| n/a | Involved in the study | |
|---|---|---|
| ☐ | ☒ | Antibodies |
| ☐ | ☒ | Eukaryotic cell lines |
| ☒ | ☐ | Palaeontology and archaeology |
| ☐ | ☒ | Animals and other organisms |
| ☒ | ☐ | Human research participants |
| ☒ | ☐ | Clinical data |
| ☒ | ☐ | Dual use research of concern |

## Methods

| n/a | Involved in the study | |
|---|---|---|
| ☒ | ☐ | ChIP-seq |
| ☒ | ☐ | Flow cytometry |
| ☒ | ☐ | MRI-based neuroimaging |

## Antibodies

| | |
|---|---|
| Antibodies used | anti-UCP1 (Made in house) |
| Validation | The anti-UCP1 antibody has been extensively used in our lab. We have performed several immuno-blots that show the antibody detects a singular band of at the correct molecular weight for UCP1 protein - 33kDa. Additionally, as shown in the supplementary figures, there is no band in adipocytes isolated from UCP1 KO animals. |

## Eukaryotic cell lines

Policy information about cell lines

| | |
|---|---|
| Cell line source(s) | Institut de Biologie Valrose (iBV) - Université Nice Sophia Antipolis - Faculté de médecine |
| Authentication | The cell line used was not authenticated |
| Mycoplasma contamination | The cell line was free of mycoplasma and virus contamination |
| Commonly misidentified lines (See ICLAC register) | None |

## Animals and other organisms

Policy information about studies involving animals; ARRIVE guidelines recommended for reporting animal research

| | |
|---|---|
| Laboratory animals | 3-4 week old Male NMRI (Han) and wild-type and UCP1 KO mice on a C57BL6 genetic background were used for adipocyte isolations |
| Wild animals | The study did not involve wild animals |
| Field-collected samples | The study did not involve field-collected samples |
| Ethics oversight | All experiments were approved by the Animal Ethics Committee of the North Stockholm region (DNR 15083-2020). |

Note that full information on the approval of the study protocol must also be provided in the manuscript.
